# Lithium in Drinking Water and Incidence of Suicide: A Nationwide Individual-Level Cohort Study with 22 Years of Follow-Up

**DOI:** 10.3390/ijerph14060627

**Published:** 2017-06-10

**Authors:** Nikoline N. Knudsen, Jörg Schullehner, Birgitte Hansen, Lisbeth F. Jørgensen, Søren M. Kristiansen, Denitza D. Voutchkova, Thomas A. Gerds, Per K. Andersen, Kristine Bihrmann, Morten Grønbæk, Lars V. Kessing, Annette K. Ersbøll

**Affiliations:** 1 National Institute of Public Health, University of Southern Denmark, Øster Farimagsgade 5A, 2nd Floor, 1353 Copenhagen, Denmark; nikoline_nk@hotmail.com (N.N.K.); akri@si-folkesundhed.dk (K.B.); mg@si-folkesundhed.dk (M.G.); 2 Geological Survey of Denmark and Greenland (GEUS), Department of Groundwater and Quaternary Geology Mapping, C.F. Møllers Allé 8, Aarhus, 8000 Aarhus, Denmark; jsc@geus.dk (J.S.); bgh@geus.dk (B.H.); geoddv@nus.edu.sg (D.D.V.); 3 Department of Public Health, Aarhus University, Bartholins Allé 2, 8000 Aarhus, Denmark; 4 National Centre for Register-Based Research, Aarhus University, Fuglesangs Allé 4, 8210 Aarhus, Denmark; 5 Geological Survey of Denmark and Greenland (GEUS), Hydrological Department, Øster Voldgade 10, 1350 Copenhagen, Denmark; lfj@geus.dk; 6 Department of Geoscience, Aarhus University, Høegh-Guldbergs Gade 2, 8000 Aarhus, Denmark; smk@geo.au.dk; 7 Current affiliation (DDV): Department of Geography, National University of Singapore, AS2, #03-01, 1 Arts Link, Kent Ridge, 117570 Singapore, Singapore; 8 Section of Biostatistics, Department of Public Health, University of Copenhagen, Øster Farimagsgade 5, 1014 Copenhagen, Denmark; tag@biostat.ku.dk (T.A.G.); pka@biostat.ku.dk (P.K.A.); 9 Psychiatric Center Copenhagen, Rigshospitalet, University Hospital of Copenhagen, Blegdamsvej 9, 2100 Copenhagen, Denmark; Lars.Vedel.Kessing@regionh.dk

**Keywords:** drinking water, lithium, suicide, individual-level data, spatial analysis, Denmark, exposure assessment

## Abstract

Suicide is a major public health concern. High-dose lithium is used to stabilize mood and prevent suicide in patients with affective disorders. Lithium occurs naturally in drinking water worldwide in much lower doses, but with large geographical variation. Several studies conducted at an aggregate level have suggested an association between lithium in drinking water and a reduced risk of suicide; however, a causal relation is uncertain. Individual-level register-based data on the entire Danish adult population (3.7 million individuals) from 1991 to 2012 were linked with a moving five-year time-weighted average (TWA) lithium exposure level from drinking water hypothesizing an inverse relationship. The mean lithium level was 11.6 μg/L ranging from 0.6 to 30.7 μg/L. The suicide rate decreased from 29.7 per 100,000 person-years at risk in 1991 to 18.4 per 100,000 person-years in 2012. We found no significant indication of an association between increasing five-year TWA lithium exposure level and decreasing suicide rate. The comprehensiveness of using individual-level data and spatial analyses with 22 years of follow-up makes a pronounced contribution to previous findings. Our findings demonstrate that there does not seem to be a protective effect of exposure to lithium on the incidence of suicide with levels below 31 μg/L in drinking water.

## 1. Introduction

Suicide is a serious public health problem accounting for 1.4% of all deaths in 2012 worldwide [1]. In Denmark, around 600 people, corresponding to 1% of all deaths, commit suicide every year since 2005 [2]. For comparison, this number is more than three times higher than the number of deaths due to traffic accidents [3]. Apart from being a personal tragedy, suicide causes profound suffering for families, relatives, and communities [1]. Suicide is a multifactorial event caused by a complex interaction between psychosocial, genetic, and environmental factors [4]. While a wide range of psychosocial factors (such as abuse, loss, or stressful life events [5]) have been investigated in terms of their influence on suicide, fewer studies have considered the potential impact of environmental exposures.

Lithium (Li) is a naturally occurring element in drinking water mainly originating from weathering of minerals in the subsurface. Levels in drinking water vary across the world for example with levels up to 12.9 µg/L in the Aomori prefecture in Japan [6] and up to 219 µg/L in Texas [7], yet everyone is exposed to some amount [8]. In clinical practice it is well established that lithium has a mood-stabilizing and suicide-preventive effect in individuals suffering from affective disorders [9,10]. Therapeutic doses vary from 600 to 2400 mg per day [11] and are magnitudes higher than levels reported from both surface and groundwater sources of drinking water [12]. However, the low lithium exposure through drinking water occurs continuously throughout the lifespan.

Recently, a series of ecological studies [6,7,13,14,15,16,17,18] has addressed the potential effect of lithium in drinking water on suicide in the general population. Although evidence is pointing in the direction of an inverse association [19], contradictory findings have been reported in different regions and a causal relation is uncertain. An essential limitation of previous studies [6,7,13,14,15,16,17,18] is that they have been conducted at an aggregate regional level prone to the possibility of ecological fallacy, where associations may be falsely overestimated compared to those established by individual-level analysis [20]. Therefore, for the first time, we investigate the association at an individual level using prospectively collected nationwide Danish register data. The unique personal identification number and the comprehensive Danish registers enabled follow-up of the entire Danish adult population (age ≥21 years, 3.7 million individuals) over the course of 22 years. All Danish drinking water is of groundwater origin and clear geographic patterns in lithium levels have been found [21]. Individuals living in Eastern Denmark are exposed to lithium levels more than 10 times higher than individuals in Western Denmark [21]. The individual-level data allow for investigation of the effect of a long-term exposure to lithium in drinking water, while accounting for people moving residences and thereby changing exposure. In addressing issues of spatial autocorrelation (see also Helbich et al. [14]), where dependence among observations violates standard statistical techniques, spatial analyses were conducted using a Bayesian conditional autoregressive (CAR) model. Overall, the aim of the present study was to investigate the effect of naturally occurring lithium in drinking water on the incidence of suicide at an individual level, hypothesizing that lithium in drinking water has a potentially protective effect on the risk of suicide in the general population. The supposition is that people, who in periods have been exposed to a high level of lithium in their drinking water, have a stabilized mood without large fluctuations, which lowers their risk of later experiencing mood disturbances so severe that they end up taking their own life.

## 2. Materials and Methods

### 2.1. Study Design and Population

The study was based on prospectively collected data and designed as a closed cohort study with a common baseline at 1 January 1991. All health-related data were obtained from the nationwide individual-level Danish registers in the study period from 1 January 1991 to 31 December 2012. All individuals with residence in Denmark have a unique personal identification number given at birth or immigration and registered in the Danish Civil Registration System (CRS) [22]. Linkage of individual-level data is possible through this unique personal identification number used in the different registers [23]. The study population included all 3,740,113 Danish adults (≥21 years) in the study period. Suicides were identified through the Danish Register of Causes of Death [24]. The International Classification of Disease, Revision 8, (ICD-8) codes used for suicide were E950-59 up to and including 1993, whereas the ICD-10 codes used from 1994 were X60–X84, Y87.0, and U03. The coverage of the population in the Danish registers is virtually complete and the validity is generally considered to be high [22,25]. Information on residential location was obtained from the CRS every year, making it possible to account for relocations and thereby changes in lithium exposure. Since data from the CRS were assessed on 1 January each year, it was not possible to obtain information about relocations (or emigrations) (and thus changes in exposure level) occurring within the same year. Individuals who emigrated contributed with risk time until emigration. The use of register information enabled follow-up of all individuals until emigration, death, or end of the study period [23]. The coordinates of the locations are given by the universal transverse mercator projection UTM EUREF89, Zone 32N.

### 2.2. Lithium Measurements

Denmark covers approximately 43,000 km^2^ and drinking water supply is based on groundwater. Naturally occurring lithium is not systematically monitored in Danish drinking water. Thus, for the present study a total of 158 drinking water measurements were obtained in a dedicated sampling campaign at 151 public waterworks supplying approximately 42% of the Danish population. The majority of the measurements (*N* = 139) came from a drinking water sampling campaign, executed from April to June 2013, spatially covering the entire country [21,26]. Analyses of lithium concentrations in the samples were performed at the Geological Survey of Denmark and Greenland (GEUS) (details can be found in [21,26]). Additional measurements (*N* = 19) came from a separate campaign at the Greater Copenhagen Utility (HOFOR) in the period October 2009 to June 2010. These samples were collected by trained HOFOR staff and analyzed by a laboratory accredited by the Danish Ministry of the Environment. Concentrations of lithium were in all samples measured by inductively-coupled plasma mass spectrometry (ICP-MS). Seven waterworks had measurements from both campaigns in which case the mean of the measurements from the two sampling campaigns was used and the results employed to verify compliance. This rendered a total of 151 drinking water lithium measurements used in the analyses. All lithium measurements were above the detection limit of 0.5 µg/L.

#### 2.2.1. Stability of Lithium Levels over Time

The drinking water lithium measurements were made at a single point in time (except for the measurements from the seven waterworks included in both sampling campaigns) and assumed to have remained constant over the study period. There exists only limited data on the temporal variability of lithium in drinking water in Denmark as well as in other countries. However, based on its chemical properties in aqueous solutions, lithium is considered to be a conservative compound that, due to the Li^+^ ions’ small size and strong hydration [27], is not expected to change over time or to react chemically with other substances during aeration and sand filtration at the water treatment plants, in the distribution system, or at installations. A previous comparison of the lithium measurements from the seven waterworks included in both sampling campaigns and made up to four years apart (between 2009 and 2013) suggests that the lithium levels were roughly stable over time and that there was no lithium removal or enrichment during the treatment at the waterworks [21].

The stability of the lithium levels over time was evaluated using 3682 groundwater lithium measurements collected between 1947 and 2012 and extracted from GEUS’s publicly available nationwide geo-database *Jupiter* on 23 September 2014 (www.geus.dk/UK/data-maps). The samples were collected for various monitoring purposes at specific depths and in specific geological layers where lithium is present in different minerals with varying concentrations. Samples collected for monitoring of contaminated sites and tracer test experiments were omitted. The groundwater lithium measurements are not representative of drinking water levels; however, since some of them comprise repeated measurements from the same location, they could be used to investigate the stability of lithium levels over time. This was done by estimating the variation in lithium concentrations between borehole, sampling-depth, and date of lithium measurement using a general linear mixed model in SAS Version 9.3. A three-level model was used with borehole, sampling-depth, and measurement date defined as random effects. There were a total of 913 boreholes, 13 sampling-depths within boreholes, and 2501 repeated measurement dates within boreholes and sampling-depths. Since the distribution of the groundwater lithium concentrations was skewed to the right, the log-transformed variable was used in the analyses. Assumptions of normality and variance homogeneity of the lithium measurements were reasonably met.

#### 2.2.2. Lithium Exposure Assessment

The lithium measurements came from 151 locations across Denmark. Kriging [28,29] was used to estimate lithium levels at locations that were not sampled based on weighted averages of surrounding measurements. A prerequisite is that lithium measurements close to each other in space are more alike than distant ones, i.e., exhibit properties of spatial autocorrelation, as found in Denmark [21]. Ordinary kriging was performed based on parameter estimates characterizing the spatial autocorrelation obtained by fitting an exponential semivariogram (i.e., range of influence, nugget, and partial sill). Before kriging was performed, the study area was converted to an image with grid cells of 1 km × 1 km. Predicted lithium values were assigned to all grid cells. In order to find the best exposure prediction, various semivariogram models were fitted and the model rendering the lowest root mean square error (RMSE) [30] was used for the lithium exposure assessment. The semivariogram and spatial interpolation of the lithium measurements were calculated in R Version 3.1.0 (R Foundation for Statistical Computing, Vienna, Austria) using the gstat package (Version 1.1.3 [31,32]).

The most detailed geographical information available for the study was municipality of residence. Therefore, the mean of the kriged lithium prediction values was calculated within each municipality rendering a lithium level for all 275 municipalities in Denmark. With the structural reform in Denmark in 2007, the 275 municipalities were merged into 98 [33]; however, municipality codes prior to 2007 were used for all years in the study period. All maps were created in R Version 3.1.0 using the sp package (Version 1.2.3 [34,35]). Based on the lithium level for the municipality of residence, exposure was calculated as a moving five-year time-weighted average (TWA) lithium exposure level (µg/L) for all individuals in the study population from 1991 until censoring or 2012. The lithium exposure level was updated each year during the follow-up period thereby accounting for people moving address and thus changing exposure level. Although lithium, due to its water solubility, does not biologically accumulate in the body [36], a long-term exposure can provide information about the effect of having been exposed to a continuously high or low level of lithium or a combination of high and low levels. The hypothesis is that people who in periods have been exposed to a high level of lithium in their drinking water have a stabilized mood without large fluctuations, thus lowering their risk of suicide later in life.

### 2.3. Spatial Regression Analysis

The effect of the five-year TWA lithium exposure level in drinking water on suicide rate was evaluated using a regression model with a Poisson distribution of the number of suicides as the outcome and logarithmic transformation of follow-up time as the offset. A random effect was included in the model to account for spatial autocorrelation, i.e., the fact that neighboring observations (i.e., municipalities in the present study) tend to be more alike compared to those farther apart. Thereby the analysis enabled the evaluation of the effect of lithium exposure on suicide rate, when accounting for the spatial autocorrelation in suicide rates between neighboring municipalities after adjustment for confounders [37]. The random effect was modelled using the conditional autoregressive (CAR) model suggested by Besag–York–Mollié (BYM) [38]. Spatial correlation between neighboring municipalities was modelled by a binary 275 × 275 neighborhood (adjacency) matrix, whose *jk*-th element is 1 if the municipalities *j* and *k* share a common border and otherwise 0. Islands with only one municipality were linked to a municipality on the nearest larger island or to the peninsula to which they are connected by a bridge or a ferry.

Follow-up time was split by calendar year resulting in approximately constant suicide rates. The incidence rate ratio (IRR) of suicide was calculated for the five-year TWA lithium exposure levels in five groups compared with the highest exposure level chosen as the reference group.

Potential confounders were selected based on the literature on central risk factors for suicide combined with a hypothesized association with lithium exposure, as well as findings from the reviewed literature on factors of influence on the association [6,7,13,14,15,16,17,18]. Covariates were included at an individual level and comprised gender and ethnicity (Danish origin, immigrant/descendant) as time constant confounders and age (10-year categories), employment (employed, unemployed, outside labor force) and civil status (cohabiting, living alone) as time-varying confounders. Additionally, since the suicide rate decreased over the course of the study period, analyses were adjusted for calendar year (five-year categories). Information on the covariates was updated every year at the individual level and came from the CRS [22] and the Employment Classification Module [25]. Due to a small number of immigrants/descendants, adjustment for ethnicity was not possible and the variable was only included in the descriptive statistics, not in the regression model.

Bayesian inference (parameter estimation) was made using integrated nested Laplace approximation (INLA) [38,39], which reduces the computation time considerably as compared to using Markov chain Monte Carlo. Gaussian hyper prior distributions were used for intercept and fixed effects with default values ((0, 0) for the intercept and (0, 0.001) for the fixed effects). Log-gamma hyper prior distributions were used for the heterogeneity and the spatial structure components with default values ((1, 0.0005) and (1, 0.0005), respectively). The effect of the selected prior distributions on the parameter estimates was evaluated by changing the default values (changed to (1, 0.01) and (1, 0.001), respectively). The spatial regression analyses were performed in R Version 3.2.2 (R Foundation for Statistical Computing, Vienna, Austria) using the INLA package [40].

### 2.4. Supplementary Analyses

Six supplementary analyses were conducted. The first supplementary analysis evaluated a 10-year TWA lithium exposure level as an alternative to the five-year TWA lithium exposure level. In the second supplementary analysis, the capital area of Copenhagen was excluded, since drinking water for this area is supplied by several waterworks outside the capital area and mixed before being distributed to the consumers. Lithium exposure in the capital area of Copenhagen might be uncertain due to this mixture of drinking water. The third supplementary analysis examined the association using a semi-adjusted model adjusted for differences in age, gender, and calendar year. In the fourth supplementary analysis, a non-spatial Poisson regression analysis of suicide incidence rates without the random effect of municipality was conducted for comparison. This analysis was completed using maximum likelihood estimation in SAS Version 9.3 (SAS Institute Inc., Cary, NC, USA). The fifth and sixth supplementary analyses were performed changing the study design from a cohort study to a matched case-control study design and an ecological study, respectively. These analyses were performed to evaluate the potential effect of the selected study design on the association between lithium exposure and incidence of suicide. A sex- and age-matched case-control study design was used, with selection of 10 controls per case by incidence density sampling, analyzed using a Cox regression model [41]. An ecological study was designed by aggregating numbers of suicides per municipality for a five-year period and analyzed using logistic regression with lithium level in the municipalities as exposure.

## 3. Results

The stability of lithium levels in groundwater over time was evaluated (Table 1). The results show that most of the variance in the lithium measurements was due to the anticipated variation between boreholes (74.7%) and between different sampling-depths within the same borehole (16.9%). The differences between measurements over time, i.e. the variation in lithium levels within the same borehole and sampling-depth on different days (residual variance 0.07), accounted for 8.4% of the total variation in the groundwater lithium measurements. As this is considered a relatively small proportion, the results of the analysis indicated that the lithium levels in Danish groundwater were reasonably stable over time.

**Figure 1:**
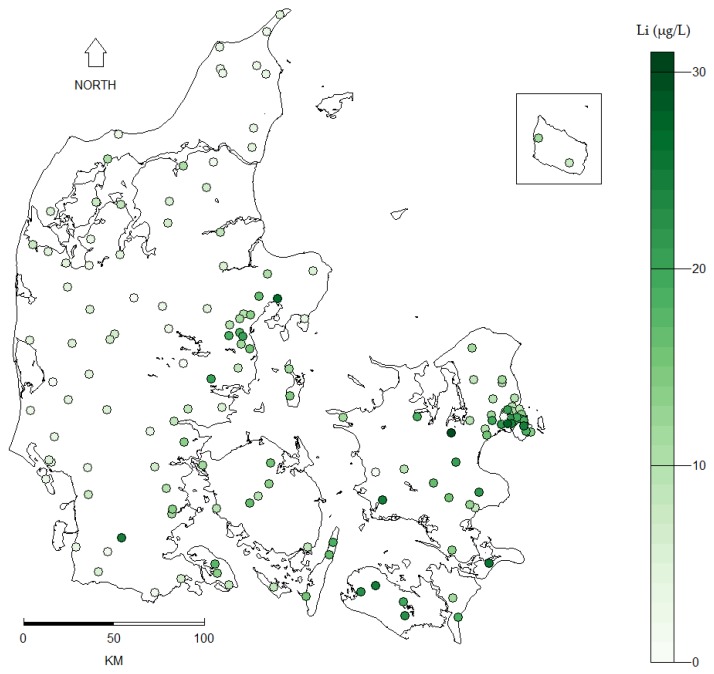
Level and geographic distribution of 151 drinking water lithium (Li) measurements sampled at Danish waterworks between 2009 and 2013.

**Figure 2:**
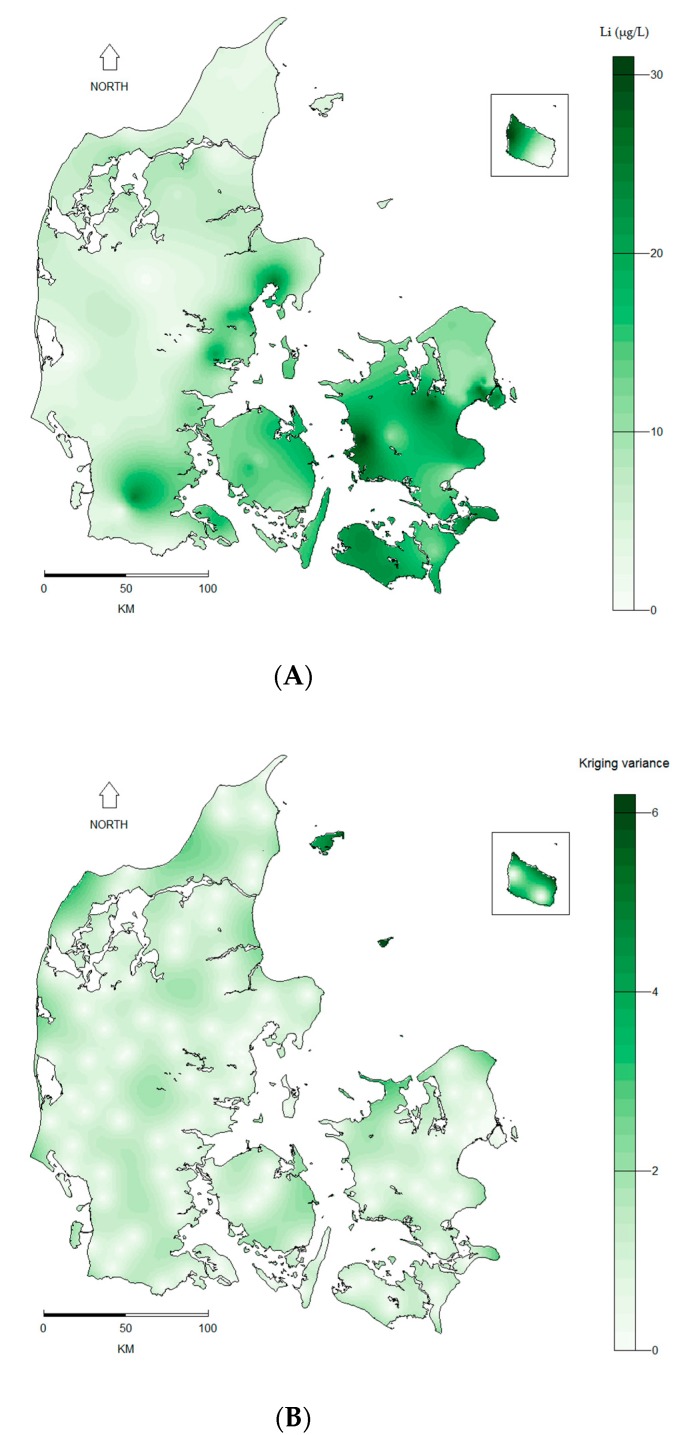
Prediction (**A**) and prediction variance (**B**) of drinking water lithium (Li in μg/L) levels based on 151 lithium measurements sampled at Danish waterworks between 2009 and 2013. Interpolation was done by kriging. Spatial interpolation of the lithium measurements was performed in R Version 3.1.0 using the gstat package (Version 1.1.3 [31,32]).

**Figure 3:**
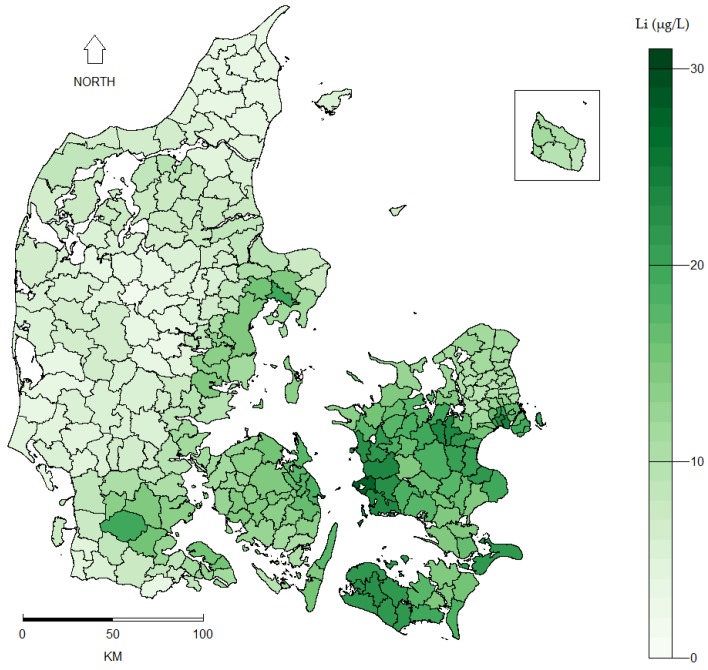
Mean municipality lithium (Li in μg/L) levels in Danish drinking water based on interpolation of 151 measurements (Figure 2A) sampled at Danish waterworks between 2009 and 2013.

The mean lithium level of the 151 drinking water measurements was 11.6 µg/L (standard deviation (SD): 6.8 µg/L) ranging from 0.6 µg/L in Western Denmark to 30.7 µg/L in Eastern Denmark (Figure 1). The interpolation of the point data done by kriging rendered a prediction map and a corresponding prediction variance (i.e., error) map (Figure 2). Similar prediction maps were found for all kriging models regardless of model specifications. Inverse distance weighting (IDW) was used as an alternative spatial interpolation method to validate the prediction obtained by kriging and rendered equivalent results. The mean drinking water lithium level for each of the 275 municipalities (based on the prediction map in Figure 2A) is shown in Figure 3.

Baseline characteristics of the study population on 1 January 1991 are shown in Table 2 by five-year TWA lithium exposure levels.

The study population consisted of a total of 3,740,113 individuals aged 21 years or older of which 51.5% were women. The cohort had a total of 66,813,931 person-years at risk during the study period. A total of 14,151 individuals, equivalent to 0.38% of the study population, committed suicide during the study period. The suicide rate decreased during the study period from 29.7 per 100,000 person-years in 1991 to 18.4 per 100,000 person-years in 2012. The suicide rates at the municipality level ranged from 6.1 to 36.0 suicides per 100,000 person-years at risk.

**Figure 4:**
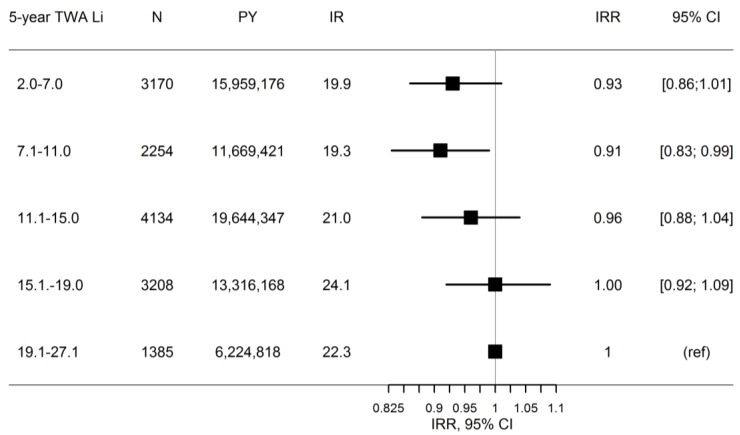
Incidence rate ratio (IRR) for suicide with increasing five-year time-weighted average (TWA) lithium exposure level (Li in μg/L) in drinking water using a Poisson regression model with the random effect modelled using a conditional autoregressive model (CAR) adjusted for differences in gender, age, employment, civil status, and calendar year. N: number of suicides; PY: person-years at risk; IR: crude incidence rate; IRR: adjusted incidence rate ratio; 95% CI: 95% credible interval; ref: reference group. The spatial regression analysis was performed in R Version 3.2.2 using the INLA package [40].

The spatial regression analysis showed no protective effect of five-year TWA lithium exposure level through drinking water on suicide rate adjusted for differences in gender, age, employment, civil status, and calendar year (Figure 4). The results indicated an increasing suicide rate by increasing five-year TWA lithium exposure level. Estimates of the covariates are shown in the Supplementary Materials, Table S1.

The supplementary analyses overall showed the same results as the main spatial regression analysis (Supplementary Materials, Table S2). Repeating the main analysis with the 10-year TWA lithium exposure level showed similar results with no protective effect of an increasing five-year TWA lithium exposure level on suicide rate. The analysis excluding the capital area of Copenhagen, the semi-adjusted analysis and the non-spatial analysis showed the same overall trend as the spatial regression analysis. Changing the study design to a matched case-control study or to an ecological study showed similar results with no protective effect of an increasing five-year TWA lithium exposure level on the incidence of suicide.

## 4. Discussion

Our study is the first to investigate the effect of naturally occurring lithium in drinking water on the incidence of suicide at an individual level with more than 20 years of follow-up. The comprehensiveness of our data and analyses makes a pronounced contribution to previous findings [19] and demonstrates that there does not seem to be a protective effect of exposure to low levels of lithium on the incidence of suicide with lithium levels below 30 μg/L in drinking water.

The main advance of our study compared to previous ecological studies, is the use of prospectively collected individual-level data following the entire Danish adult population over the course of 22 years. The use of these data avoided selection bias [42] and enabled computation of a five-year TWA lithium exposure level accounting for people moving residence and thereby changing exposure during the study period. Calculation of a five-year TWA lithium exposure level based on a one-time lithium measurement was possible as lithium levels were found to be stable over time when analyzing groundwater data. Further, it was possible to incorporate updated information on all covariates each year, making the confounder adjustment more exact. This was particularly important for the adjustment for employment and civil status, since especially changes in these factors might affect the incidence of suicide.

The lithium exposure assessment in the present study was based on drinking water samples from public waterworks. This is appropriate for Denmark, since the public water supply is the main source of drinking water, and bottled water consumption is amongst the lowest in the EU (20 L per person per year) [43,44,45]. Kriging was used to estimate lithium exposure levels at locations that were not sampled. Kriging is an interpolation method developed for continuous variables. Stationarity is an assumption of kriging, which means that the spatial correlation structure (i.e., the semivariogram parameters) should be constant across locations within the study area. Gotway and Wolfinger showed that despite deviations from stationarity and misspecified semivariograms, the kriging estimates are relatively unbiased [46]. Maps of kriging estimates of the lithium level in drinking water in the present study were very similar for different combinations of semivariogram parameters. Spatial interpolation using IDW as a non-parametric method also resulted in a similar map. It is therefore concluded that ordinary kriging used in the present study is a suitable method for deriving a map of estimated lithium levels. The interpolation of the lithium point measurements assumes that individuals are exposed to the level of lithium found at the waterworks close to their residential location. This assumption is reasonable due to the highly decentralized water supply in most areas of Denmark [47]. This is, however, not true for the capital area of Copenhagen, where drinking water is supplied by several large waterworks outside the city and mixed before reaching the tap at the consumer. Yet, sensitivity analyses excluding individuals residing in the capital area of Copenhagen did not significantly alter the results. Since Copenhagen is the largest urban area in Denmark, the sensitivity analysis also indicated that confounding due to urbanicity is not likely to have occurred. Using actual water supply areas instead of interpolation of point measurements might increase the certainty of the lithium exposure estimation (e.g., [46]). However, this approach was not used in the present study as lithium measurements were not available from all water supply areas.

The study also has some limitations. As mentioned previously, lithium in drinking water is not monitored routinely in Denmark. The lithium measurements used in the present study were obtained in a sampling campaign of 151 public waterworks supplying approximately half the Danish population. Although temporal stability of groundwater lithium measurements was seen, measurements of lithium in drinking water would be needed from more waterworks for a number of years to examine the stability of lithium in drinking water over time. Further, drinking water is not the only source of lithium exposure as the element is also present in some amounts in food, e.g., by uptake from vegetables through the soil [8]. Addressing the issue of other potential sources of lithium intake would be relevant in future research. Additionally, it would be relevant to evaluate the potential effect of lithium prescriptions on the association, although a study from 2015 found that suicide and lithium levels in drinking water were not a function of lithium prescription rates across Austria [48].

In the present study, Danish drinking water lithium levels were found to range from 0.6 to 30.7 µg/L with a mean level of 11.6 µg/L (median 10.5 µg/L) and a standard deviation (SD) of 6.8 µg/L. Where the mean level was comparable to levels found in previous studies [6,7,13,14,15,16,17,18], the range in the present study was generally more narrow. Previous studies that found a significant association with suicide consistently reported the highest lithium exposure levels with up to 59 µg/L in the Oita prefecture in Japan [16], 121 µg/L in Greece [13], and 219 µg/L in Texas [7]. Conversely, studies with the lowest levels up to a maximum of 12.9 µg/L in the Aomori prefecture in Japan [6] and 21 µg/L in the east of England [15] did not find an association, like in our present study in Denmark. The lack of variation in lithium levels in the present study may have challenged our analyses. Nevertheless, the results from our present study, together with the studies from Japan and the east of England, show that exposure to very low levels up to 31 µg/L does not seem to have an effect on the incidence of suicide.

Lithium’s biochemical mechanisms of action are complex and not fully understood. In addition to its mood-stabilizing, antidepressive, and antimanic effects in individuals with bipolar disorder, human studies suggest that lithium in therapeutic doses has an anti-suicidal effect [10,49,50]. This may be mediated through its mood-stabilizing properties or directly through a reduction of aggressiveness and impulsivity, characteristics that are associated with an increased incidence of suicide [51]. In our present comprehensive study, we did not find that low natural levels of lithium reduced the incidence of suicide. Thus, further studies are needed to investigate whether there is an association between suicide and natural lithium exposures higher than those observed in the present study.

## 5. Conclusions

Several studies performed at an aggregate level have suggested an association between higher levels of lithium in drinking water and a reduced risk of suicide. In the present study, individual-level register-based data on the entire Danish adult population (3.7 million individuals) from 1991 to 2012 were linked with a five-year TWA lithium exposure level from drinking water. Our study is the first to investigate the effect of naturally occurring lithium in drinking water on the incidence of suicide at an individual level with long follow-up.

The mean lithium level in drinking water was 11.6 μg/L ranging from 0.6 to 30.7 μg/L. We found no significant association between increasing five-year TWA lithium exposure level and decreasing suicide rate. Our findings demonstrate that there does not seem to be a protective effect of exposure to lithium on the incidence of suicide with levels below 31 μg/L in drinking water. The comprehensiveness of using individual-level data and spatial analyses with 22 years of follow-up makes a pronounced contribution to previous findings.

## Figures and Tables

**Table 1:** Variance estimates of groundwater lithium (Li) levels between borehole, sampling-depth, and date of measurement. Variance estimates were calculated for the log transformation of the groundwater lithium levels (*N* = 3682).

Covariate	Variance Estimate (σ^2^)	Standard Error (SE)	% of Total Variance
Borehole	0.62	0.038	74.7%
Sampling-depth within borehole	0.14	0.011	16.9%
Residual ^a^	0.07	0.002	8.4%
Total	0.83		

^a^ Measurement date within same borehole and depth.

**Table 2:** Baseline characteristics of the study population on 1 January 1991 by five-year time-weighted average (TWA) lithium exposure level.

Variable	Category	N (%)	Five-Year Time-Weighted Average Lithium Exposure Level (μg/L) N (Row %)				
			2.0 to 7.0	7.1 to 11.0	11.1 to 15.0	15.1 to 19.0	19.1 to 27.1
**Overall**	-	3,740,113	870,515	635,331	1,099,608	796,428	338,231
**Gender**	Female	1,925,833 (51.5%)	441,681 (22.9%)	325,554 (16.9%)	565,959 (29.4%)	419,036 (21.8%)	173,603 (9.0%)
	Male	1,814,280 (48.5%)	428,834 (23.6%)	309,777 (17.1%)	533,649 (29.4%)	377,392 (20.8%)	164,628 (9.1%)
**Age (years)**	21 to 29	680,857 (18.2%)	148,436 (21.8%)	106,798 (15.7%)	214,690 (31.5%)	158,474 (23.3%)	52,459 (7.7%)
	30 to 39	715,654 (19.1%)	168,975 (23.6%)	117,535 (16.4%)	214,604 (30.0%)	152,048 (21.3%)	62,492 (8.7%)
	40 to 49	762,730 (20.4%)	178,197 (23.4%)	134,614 (17.7%)	226,273 (29.7%)	150,552 (19.7%)	73,094 (9.6%)
	50 to 59	538,850 (14.4%)	128,434 (23.8%)	95,146 (17.7%)	158,237 (29.4%)	102,869 (19.1%)	54,164 (10.1%)
	60 to 69	480,608 (12.9%)	114,943 (23.9%)	83,822 (17.4%)	135,738 (28.2%)	97,048 (20.2%)	49,057 (10.2%)
	70 to 79	369,255 (9.9%)	86,757 (23.5%)	64,085 (17.4%)	99,956 (27.1%)	85,957 (23.3%)	32,500 (8.8%)
	≥80	192,159 (5.1%)	44,773 (23.3%)	33,331 (17.4%)	50,110 (26.1%)	49,480 (25.8%)	14,465 (7.5%)
**Employment**	Employed	2,509,874 (67.1%)	592,316 (23.6%)	433,552 (17.3%)	746,324 (29.7%)	507,688 (20.2%)	229,994 (9.2%)
	Unemployed	232,701 (6.2%)	49,327 (21.2%)	36,262 (15.6%)	70,786 (30.4%)	53,781 (23.1%)	22.545 (9.7%)
	Outside labor force	997,538 (26.7%)	228,872 (22.9%)	165,517 (16.6%)	282,498 (28.3%)	234,959 (23.6%)	85,692 (8.6%)
**Civil status**	Cohabiting	2,524,134 (67.5%)	626,256 (24.8%)	448,595 (17.8%)	743,470 (29.5%)	468,507 (18.6%)	237,306 (9.4%)
	Living alone	1,215,979 (32.5%)	224,259 (20.1%)	186,736 (15.4%)	356,138 (29.3%)	327,921 (27.0%)	100,925 (8.3%)
**Ethnicity**	Danish origin	3,620,674 (96.8%)	857.037 (23.7%)	618,360 (17.1%)	1,063,430 (29.4%)	754,484 (20.8%)	327,363 (9.0%)
	Immigrant/descendant	119,439 (3.2%)	13,478 (11.3%)	16,971 (14.2%)	36,178 (30.3%)	41,944 (35.1%)	10,868 (9.1%)

## Supplementary Materials

The following are available online at www.mdpi.com/1660-4601/14/6/627/s1, Table S1: Incidence rate ratio (IRR) and 95% credible interval (95% CI) for suicide for covariates included in the main model (Figure 4), Table S2: Incidence rate ratio (IRR) for suicide with increasing 10-year and five-year time-weighted average (TWA) of lithium exposure level (Li in μg/L) in drinking water using a conditional autoregressive model (CAR) adjusted for differences in gender, age, employment, civil status, and calendar year .
