# Supplementary material for: Lithium in Drinking Water and Incidence of Suicide: A Nationwide Individual-Level Cohort Study with 22 Years of Follow-Up
Source: Int J Environ Res Public Health. 2017 Jun 10;14(6):627. doi: 10.3390/ijerph14060627 (PMC5486313; doi:10.3390/ijerph14060627)
Supplement: Supplementary file 1 [file ijerph-14-00627-s001.pdf]

International Journal of
Environmental Research and Public Health

MDPI

Supplementary Materials: Lithium in Drinking Water and Incidence of Suicide: A Nationwide Individual-Level Cohort Study with 22 Years of Follow-Up

Table S1. Incidence rate ratio (IRR) and 95% credible interval (95% CI) for suicide for covariates included in the main model (Figure 4).

Variable	Level	N	PY	IR	IRR	95% CI
Gender	Female	4417	34,630,926	12.8	1	(ref)
	Male	9734	32,183,005	30.2	2.65	[2.55; 2.75]
Age (years)	21–39	461	3,303,521	14.0	1	(ref)
	40–49	1647	10,257,854	16.1	1.74	[1.57; 1.93]
	50–59	3086	15,329,314	20.1	2.42	[2.19; 2.67]
	60–69	3065	14,401,288	21.3	2.48	[2.24; 2.74]
	70–79	2353	11,410,022	20.6	1.56	[1.40; 1.73]
	≥80	3539	12,111,932	29.2	1.55	[1.39; 1.73]
Employment	Employed	5528	38,858,048	14.2	0.41	[0.39; 0.43]
	Unemployed	749	2,249,784	33.3	0.85	[0.78; 0.92]
	Outside labour force	7874	25,706,099	30.6	1	(ref)
Civil status	Cohabiting	6243	45,691,500	13.7	1	(ref)
	Living alone	7908	21,122,431	37.4	2.65	[2.56; 2.75]
Calendar year	1991–1994	4162	14,426,113	28.9	2.11	[1.99; 2.25]
	1995–1999	3591	16,512,527	21.7	1.36	[1.28; 1.44]
	2000–2004	2778	14,957,265	18.6	1.11	[1.04; 1.18]
	2005–2008	1893	10,916,098	17.3	1.00	[0.93; 1.07]
	2009–2012	1727	10,001,928	17.3	1	(ref)

N: Number of suicides; PY: Person-years at risk; IR: Crude incidence rate; IRR: Adjusted incidence rate ratio; 95% CI: 95 % credible interval; ref: Reference group.

Table S2. Incidence rate ratio (IRR) for suicide with increasing 10-year and five-year time-weighted average (TWA) of lithium exposure level (Li in µg/L) in drinking water using a conditional autoregressive model (CAR) adjusted for differences in gender, age, employment, civil status, and calendar year.

Li	IRR, 95% CI											
	10-year TWA		Five-year TWA									
			Exclusive Copenhagen area		Non-spatial#		Semi-adjusted		Matched case-control#		Ecological#	
2.0–7.0	0.90	0.84; 0.98	0.89	0.83; 0.95	0.91	0.86; 0.97	0.88	0.80; 0.96	0.91	0.85; 0.96	0.90	0.81; 0.99
7.1–11.0	0.91	0.83; 0.98	0.89	0.83; 0.95	0.90	0.84; 0.96	0.88	0.80; 0.97	0.91	0.85; 0.97	0.93	0.83; 1.03
11.1–15.0	0.92	0.85; 0.99	0.93	0.87; 0.99	0.94	0.89; 1.00	0.99	0.91; 1.08	0.95	0.89; 1.00	0.95	0.87; 1.05
15.1–19.0	0.97	0.90; 1.05	1.02	0.95; 1.10	0.98	0.92; 1.04	1.06	1.97; 1.16	0.99	093; 1.05	1.08	0.98; 1.19
19.1–27.1	1	(ref)	1	(ref)	1	(ref)	1	(ref)	1	(ref)	1	(ref)

IRR: Adjusted incidence rate ratio; 95% CI: 95% credible interval (#: 95% confidence interval); ref: Reference group.
